# Phosphatase inhibition by LB-100 enhances BMN-111 stimulation of bone growth

**DOI:** 10.1172/jci.insight.141426

**Published:** 2021-05-10

**Authors:** Leia C. Shuhaibar, Nabil Kaci, Jeremy R. Egbert, Thibault Horville, Léa Loisay, Giulia Vigone, Tracy F. Uliasz, Emilie Dambroise, Mark R. Swingle, Richard E. Honkanen, Martin Biosse Duplan, Laurinda A. Jaffe, Laurence Legeai-Mallet

**Affiliations:** 1 Department of Cell Biology, University of Connecticut Health Center, Farmington Connecticut, USA.; 2 Université de Paris, Imagine Institute, Laboratory of Molecular and Physiopathological Bases of Osteochondrodysplasia, INSERM UMR 1163, F‑75015, Paris, France.; 3 Inovarion, F-75005 Paris, France.; 4 Department of Biochemistry and Molecular Biology, University of South Alabama, Mobile Alabama, USA.; 5 Service de Médecine Bucco-Dentaire, Hôpital Bretonneau, AP-HP, Paris, France.

**Keywords:** Bone Biology, Therapeutics, Bone disease, Drug therapy

## Abstract

Activating mutations in fibroblast growth factor receptor 3 (FGFR3) and inactivating mutations in the natriuretic peptide receptor 2 (NPR2) guanylyl cyclase both result in decreased production of cyclic GMP in chondrocytes and severe short stature, causing achondroplasia (ACH) and acromesomelic dysplasia, type Maroteaux, respectively. Previously, we showed that an NPR2 agonist BMN-111 (vosoritide) increases bone growth in mice mimicking ACH (*Fgfr3^Y367C/+^*). Here, because FGFR3 signaling decreases NPR2 activity by dephosphorylating the NPR2 protein, we tested whether a phosphatase inhibitor (LB-100) could enhance BMN-111–stimulated bone growth in ACH. Measurements of cGMP production in chondrocytes of living tibias, and of NPR2 phosphorylation in primary chondrocytes, showed that LB-100 counteracted FGF-induced dephosphorylation and inactivation of NPR2. In ex vivo experiments with *Fgfr3^Y367C/+^* mice, the combination of BMN-111 and LB-100 increased bone length and cartilage area, restored chondrocyte terminal differentiation, and increased the proliferative growth plate area, more than BMN-111 alone. The combination treatment also reduced the abnormal elevation of MAP kinase activity in the growth plate of *Fgfr3^Y367C/+^* mice and improved the skull base anomalies. Our results provide a proof of concept that a phosphatase inhibitor could be used together with an NPR2 agonist to enhance cGMP production as a therapy for ACH.

## Introduction

Achondroplasia (ACH), the most common form of dwarfism, is due to a gain-of-function mutation in the fibroblast growth factor receptor type 3 (*FGFR3*) gene (1, 2). FGFR3 is expressed in growth plate cartilage and bone, which explains the bone anomalies observed in patients with ACH. The characteristic features of these patients are short arms and legs, macrocephaly, hypoplasia of the midface, lordosis, foramen magnum stenosis, and spinal compression (3). The generation of *Fgfr3*-specific mouse models has highlighted the role of FGFR3 during bone growth. In the absence of *Fgfr3*, the most prominent phenotype of the mice is overgrowth, thus indicating that FGFR3 is a negative regulator of bone growth (4, 5). Conversely, mice expressing a *Fgfr3*-activating mutation develop dwarfism and have reduced linear growth and impaired endochondral ossification, with reduced chondrocyte proliferation and reduced hypertrophic differentiation (6–10). A complex intracellular network of signals, including FGFR3, mediates this skeletal phenotype. Activating mutations in FGFR3 lead to upregulated FGFR3 protein (11) and to increased activity of several downstream intracellular signaling pathways, including MAPK, PI3K/AKT, PLCγ, and STATs (12).

During development, the rate of longitudinal bone growth is determined by chondrocyte proliferation and differentiation and is regulated by several secreted growth factors and endocrine factors, including parathyroid hormone-like peptide, Indian Hedgehog, bone morphometric proteins, transforming growth factor β, insulin like growth factor, and C-type natriuretic peptide (CNP, ref. 13). CNP and its receptor, the guanylyl cyclase natriuretic peptide receptor 2 (NPR2, also known as guanylyl cyclase B), are expressed in chondrocytes as well as in osteoblasts and are recognized as important regulators of longitudinal bone growth and bone homeostasis. NPR2 possesses guanylyl cyclase activity that leads to synthesis of cyclic guanosine monophosphate (cGMP), and dysregulation of this pathway is responsible for skeletal disorders. In clinical studies, inactivating mutations of *NPR2* were found to cause a rare form of extreme short stature, called acromesomelic dysplasia, type Maroteaux (14–16). Conversely, heterozygous NPR2 gain-of-function mutations cause tall stature (17), and overexpression of CNP due to a balanced translocation is responsible for overgrowth and bone anomalies (18, 19). Mouse models with *Npr2* loss-of-function mutations or with disruption of the CNP gene (*Nppc*) also show severe dwarfism (20–24), and an *Npr2* gain-of-function mutation causes overgrowth (25). All of these data support a key role of the CNP/NPR2 signaling pathway for normal growth.

Previous studies have indicated that, among its diverse signaling effects, activation of FGFR3 results in reduced phosphorylation and activity of NPR2 in the growth plate (26, 27). Because CNP activation of NPR2 requires that the receptor is phosphorylated on multiple serines and threonines (28, 29), FGF-induced NPR2 dephosphorylation lowers cGMP and opposes bone growth. The significance of this aspect of FGF signaling for ACH was definitively established by the recent finding that, in a mouse model of ACH, bone growth is restored by replacing the NPR2 protein with a dephosphorylation resistant form of NPR2 (*NPR2*^7E/7E^, also known as GC-B^7E/7E^) with a modified version of the protein that cannot be dephosphorylated (30). Treatment with CNP or a protease-resistant CNP analog, known as BMN-111 or vosoritide, also increases bone growth in mouse models of ACH (31, 32), and BMN-111 is currently in clinical development, with phase 2 and 3 results showing additional height gain in ACH patients (33, 34). These accumulating results, together with evidence that a PPP-family phosphatase mediates the FGF-induced dephosphorylation and inactivation of NPR2 (26, 27), suggest that a PPP-family phosphatase inhibitor could enhance bone growth in ACH patients if applied together with a CNP analog.

Here, we tested this concept using a semiselective PPP family phosphatase inhibitor, LB-100 (35). In studies of animal cancers, LB-100 has been shown to enhance responses to immunotherapy, CAR T cell therapy, and tyrosine kinase inhibitors (36–38). Phase 1 clinical trials concluded that the safety, tolerability and preliminary evidence of antitumor activity supported continued testing as a potentially novel treatment for human cancers (39). Here, we find that LB-100 counteracts the FGF-induced dephosphorylation and inactivation of NPR2, complementing the CNP stimulation and promoting bone growth in a mouse model of ACH. Our results provide evidence for the concept that an inhibitor of NPR2 dephosphorylation could be used together with an NPR2 agonist to enhance cGMP production as a therapy for ACH.

## Results

### LB-100 counteracts the inactivation of Npr2 by FGF in growth plate chondrocytes.

**Figure 1:**
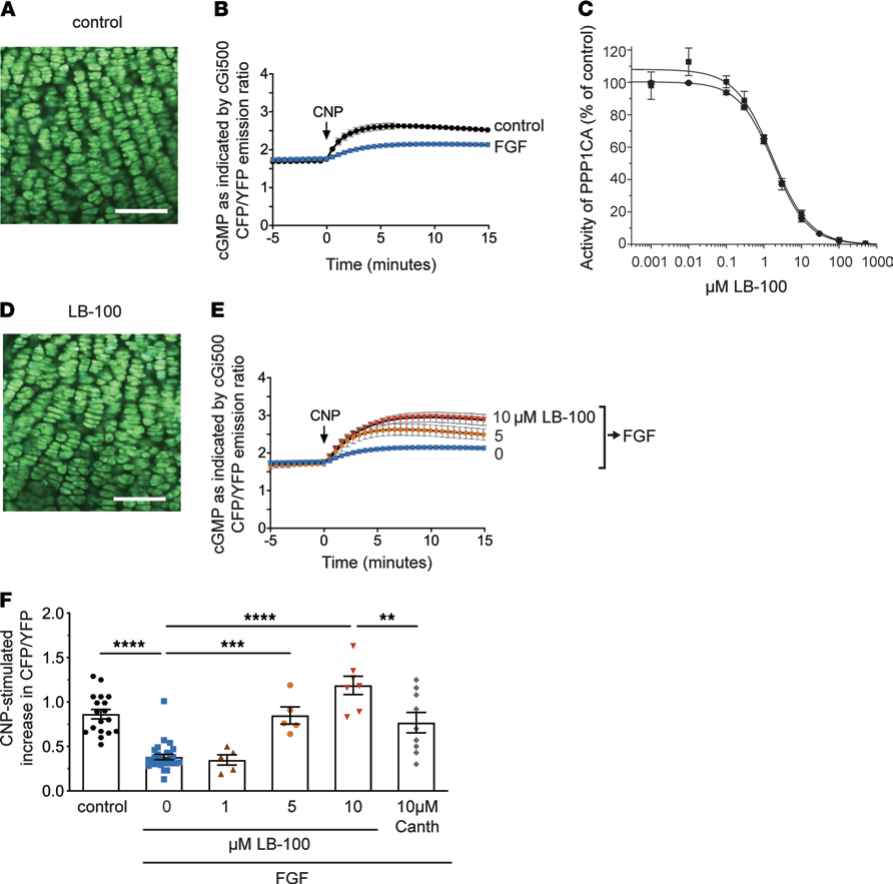
LB-100 counteracts the inactivation of NPR2 by FGF in growth plate chondrocytes of intact tibias from newborn mice. (**A**) Confocal image of cGi500 fluorescence in chondrocytes in the columnar and prehypertrophic region of the tibia growth plate, as used for measurements of cGMP production. Scale bar: 100 μm. (**B**) cGMP increase in growth plate chondrocytes in response to perfusion of 0.1 μM CNP, and inhibition of the cGMP increase by pretreatment with 0.5 μg/mL FGF18 + 1 μg/mL heparin for 80 minutes. Control tibias were pretreated for 80 minutes with heparin only. The graph shows the mean ± SEM for 27 control tibias and 18 FGF-treated tibias. (**C**) Inhibitory effect of LB-100 on the activity of recombinant PPP1CA by LB-100 assayed using DiFMUP (diamonds) or [^32^P]-labeled histone (squares) as a substrate. Each point represents the mean ± SD (*n =* 3–4). IC_50_ values are provided in Table 1. (**D**) Confocal image of cGi500 fluorescence in growth plate chondrocytes after pretreatment with 10 μM LB-100 for 2 hours. No difference in morphology was seen compared with control tibias (**A**) without LB-100. Scale bar: 100 μm. (**E** and **F**) Effect of LB-100 (or cantharidin) preincubation on CNP-stimulated cGMP production in FGF-treated tibias. Tibias expressing cGi500 were preincubated with solutions with or without LB-100 for 60 minutes. FGF was then added, and 80 minutes later, tibias were placed in a perfusion slide for cGi500 imaging during CNP perfusion. (**E**) The CFP/YFP emission ratio as a function of time after CNP perfusion. (**F**) The CFP/YFP emission ratio at 15 minutes after CNP perfusion. Symbols indicate individual tibias (*n =* 5–27). For **E** and **F**, data are shown as mean ± SEM. Data were analyzed by 1-way ANOVA followed by the Holm-Sidak multiple comparison test. **P < 0.01, ***P < 0.001, ****P < 0.0001.

NPR2 activity in chondrocytes of intact growth plates was measured as previously described, using mice expressing a FRET sensor for cGMP, cGi500 (27). The mice were WT for *Fgfr3*. Tibias were isolated from newborn mice, and the overlying tissue was excised to expose the growth plate for confocal imaging (Figure 1A). When the NPR2 agonist CNP was perfused across the growth plate, the CFP/YFP emission ratio from cGi500 increased, indicating an increase in cGMP, due to stimulation of the guanylyl cyclase activity of NPR2 (Figure 1B). Similar results were obtained with protease-resistant BMN-111 (Supplemental Figure 1; supplemental material available online with this article; https://doi.org/10.1172/jci.insight.141426DS1). Perfusion of A-type natriuretic peptide (ANP), which activates the NPR1 guanylyl cyclase, or perfusion of a nitric oxide donor (DEA/NO), which activates soluble guanylyl cyclases, did not increase cGMP (Supplemental Figure 2), showing that — among the several mammalian guanylyl cyclases — only NPR2 is active in the chondrocytes of the mouse growth plate. As previously shown (27), exposure of the growth plate to FGF18 suppressed the cGMP increase in response to CNP perfusion (Figure 1B), indicating that FGF receptor activation decreases NPR2 activity.

Based on previous evidence that a PPP-family phosphatase inhibitor, cantharidin (100 μM), inhibits the inactivation of NPR2 in growth plate chondrocytes by FGF (27), we tested whether a less toxic cantharidin derivative, LB-100, would increase NPR2 activity and long bone growth. LB-100 was originally reported as a specific inhibitor of the catalytic subunit of PPP2 (PPP2C) but was later shown to also act as a catalytic inhibitor of PPP5C (35). Since cantharidin demonstrates only modest selectivity for PPP2C versus PPP1C (40), we tested the ability of LB-100 to inhibit PPP1C activity using 2 established assays that use different substrates. We determined that LB-100 also inhibits PPP1C with an IC_50_ < 2 μM (Figure 1C and Table 1). Based on its structural similarity with cantharidin, 10 μM LB-100 is not likely to inhibit PPP3C/calcineurin or PPP7C/PPPEF (41).

To investigate if LB-100 counteracts the inactivation of NPR2 by FGF, we preincubated the tibia with or without LB-100 and then with FGF. Following these incubations, the tibia was placed in a perfusion slide for confocal imaging, and cGMP production by NPR2 was monitored by measuring the increase in the CFP/YFP emission ratio in response to CNP. The 2-hour incubation with 10 μM LB-100 caused no visible change in chondrocyte morphology, as imaged in the live growth plate (compare Figure 1D with the control in Figure 1A).

After FGF treatment, the cGMP increase in response to CNP was small (Figure 1E). However, when the tibia was preincubated with 5 or 10 μM LB-100 before applying FGF, the CNP-induced cGMP increase was enhanced (Figure 1, E and F). A concentration of 1 μM LB-100 had no effect (Figure 1F). The CFP/YFP emission ratio attained after CNP perfusion in tibias that had been incubated in 5 or 10 μM LB-100 before the FGF treatment was similar to or greater than the ratio in control tibias without FGF (Figure 1F). Figure 1F summarizes the CNP-stimulated increases in the CFP/YFP emission ratio from cGi500 under these various conditions and demonstrates that LB-100 counteracts the inactivation of NPR2 by FGF. LB-100 was more effective than cantharidin, with 5 μM LB-100 resulting in a stimulation equivalent to that seen with 10 μM cantharidin (Figure 1F).

### LB-100 counteracts the FGF-induced dephosphorylation of NPR2 by FGF in primary chondrocyte cultures.

To investigate if LB-100 counteracts the FGF-induced dephosphorylation of NPR2, we used Phos-tag gel electrophoresis (42) to analyze the phosphorylation state of NPR2 in isolated chondrocytes from the ribs of newborn mice. The mice were WT for *Fgfr3*. To allow specific labeling of the NPR2 protein, the mice were genetically modified to insert a 9–amino acid hemagglutinin (HA) tag on the N-terminus of NPR2 (HA-*Npr2*; ref. 43) (Supplemental Figure 3). We compared the phosphorylation state of NPR2 in chondrocytes with and without LB-100 preincubation — and with and without subsequent exposure to FGF. Treated and untreated chondrocytes had a similar appearance (Supplemental Figure 4).

**Figure 2:**
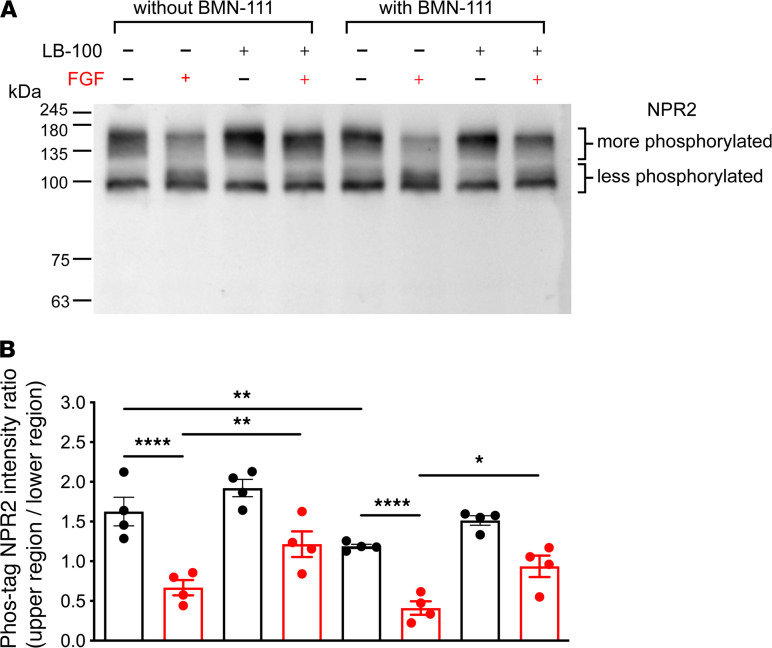
LB-100 counteracts the FGF-induced dephosphorylation of NPR2 in primary chondrocyte cultures. (**A**) Western blot of a Phos-tag gel showing the decrease in NPR2 phosphorylation in chondrocytes treated with FGF18 (0.5 μg/mL) for 10 minutes and attenuation of this dephosphorylation in chondrocytes pretreated with LB-100 (10 μM) for 1-hour prior to treatment with FGF18 (lanes 1–4). Lanes 5–8 show samples that were treated similarly, except that the protease-resistant CNP analog BMN-111 (0.1 μM) was present during the 1-hour pretreatment period. Chondrocytes were obtained from mice in which the endogenous NPR2 was tagged with an HA epitope. Blots were probed with an antibody recognizing the HA epitope. (**B**) Densitometry measurements for 4 experiments like that shown in **A**. The *y* axis indicates the ratio of the intensity of the upper region to that of the lower region as shown in **A**; a smaller ratio indicates a decrease in NPR2 phosphorylation. Symbols indicate individual experiments; data are shown as mean ± SEM. Data were analyzed by repeated measures 2-way ANOVA followed by Holm-Sidak multiple comparisons tests between the indicated groups. *P < 0.05; **P < 0.01; ****P < 0.0001.

Chondrocyte proteins were separated by Phos-tag gel electrophoresis, which slows migration of phosphorylated proteins, and Western blots were probed for NPR2 (Figure 2A). Without FGF treatment, NPR2 protein from the rib chondrocytes was present in a broad region of the gel. With FGF treatment, the ratio of the signal in the upper versus lower regions decreased (Figure 2, A and B; compare lanes 1 and 2), indicating NPR2 dephosphorylation in response to FGF and confirming, with primary chondrocytes, a previous study using a rat chondrosarcoma (RCS) cell line (26). However, if the chondrocytes were preincubated with 10 μM LB-100, the dephosphorylation in response to FGF was only partial, indicating that LB-100 counteracts the FGF-induced dephosphorylation of NPR2 (Figure 2, A and B; compare lanes 2 and 4).

To more closely mimic conditions used in experiments to be described below, the NPR2 phosphorylation state was also analyzed using chondrocyte cultures to which we added the protease-resistant BMN-111. The addition of BMN-111 caused some reduction in NPR2 phosphorylation (Figure 2, A and B; compare lanes 1 and 5), independently of treatment with FGF (Figure 2, A and B; compare lanes 2 and 6). This is consistent with previous evidence that some NPR2 dephosphorylation occurs in response to prolonged agonist (CNP) exposure (28). However, the addition of BMN-111 did not change the conclusions that FGF causes NPR2 dephosphorylation (Figure 2, A and B; compare lanes 5 and 6) and that LB-100 counteracts the FGF-induced dephosphorylation (Figure 2, A and B; compare lanes 6 and 8).

### In Fgfr3^Y367C/+^ femurs, LB-100 enhances the stimulation of bone growth by the protease-resistant NPR2 agonist BMN-111.

**Figure 3:**
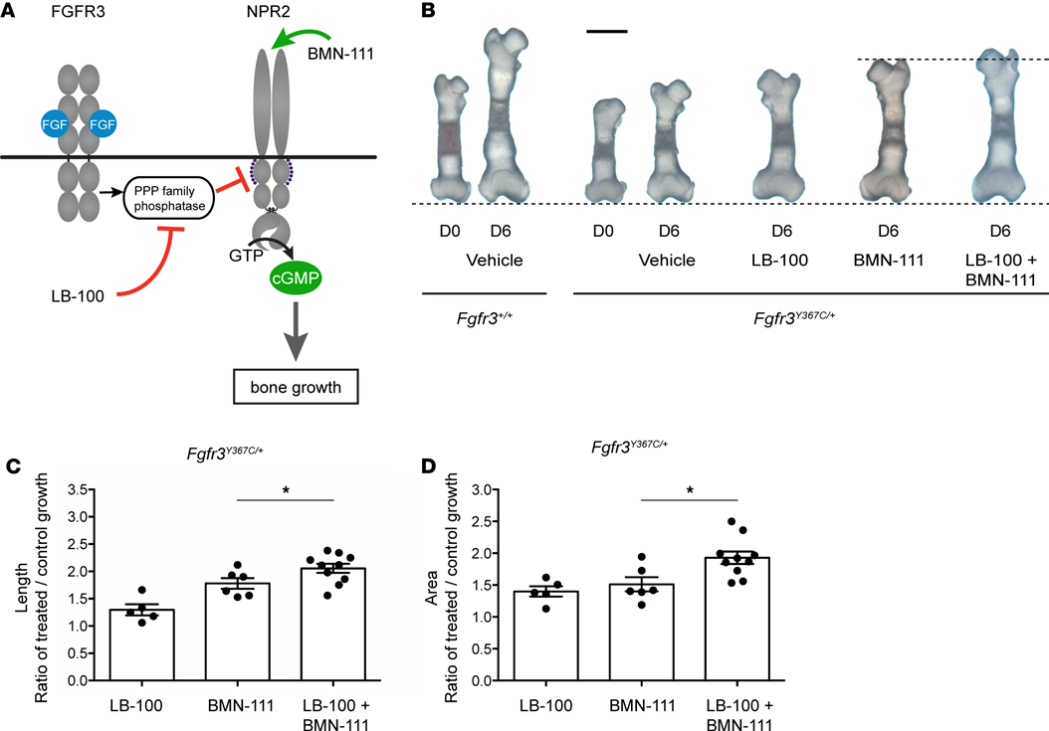
LB-100 and BMN-111 act synergistically to stimulate growth in fetal femurs from *Fgfr3^Y367C/+^* mice. (**A**) Diagram showing sites of action of LB-100 and BMN-111. (**B**) Representative photographs of fetal femurs from E16.5 *Fgfr3*^+/+^ (WT) and *Fgfr3^Y367C/+^* mice, before (D0) and after a 6-day (D6) culture with the indicated treatments. The upper dashed line indicates the groups compared in **C** and **D**. Scale bar (solid line): 1 mm. (**C** and **D**) Measurements of growth in bone length (**C**) and area (**D**), showing that LB-100 + BMN-111 increases growth more than BMN-111 alone. The concentration of LB-100 was 10 μM, and the concentration of BMN-111 was 0.1 μM. Symbols in graphs **C** and **D** indicate individual bones (*n =* 5–10). Data are shown as mean ± SEM, and data were analyzed by unpaired 2-tailed *t* tests. *P < 0.05 between indicated groups.

Previously, we showed that the protease-resistant CNP analog BMN-111 increases bone growth in a mouse model of ACH, in which tyrosine 367 is changed to a cysteine (*Fgfr3^Y367C/+^*), resulting in constitutive activation of FGFR3 (32, 44). However, BMN-111 only partially rescued the effect of the FGFR3-activating mutation. Our finding that LB-100 opposes the FGF-induced dephosphorylation and inhibition of NPR2 activity in chondrocytes suggested that applying LB-100 together with BMN-111 might enhance the stimulation of growth in bones from *Fgfr3^Y367C/+^* mice (Figure 3A).

As previously reported (32), 0.1 μM BMN-111 increased the growth of cultured femurs from E16.5 *Fgfr3^Y367C/+^* mice (Figure 3, B–D). Over a 6-day culture period, the mean increase in bone length in the BMN-111–stimulated *Fgfr3^Y367C/+^* femurs was 1.78 times that in vehicle-treated bones (Figure 3C). LB-100 alone also increased the extent of elongation, showing a growth ratio of 1.30 for LB-100/control (Figure 3C). However, when *Fgfr3^Y367C/+^* femurs were cultured with BMN-111 together with 10 μM LB-100, the mean increase in bone length was 2.06 times that in untreated bones (Figure 3C). Thus, the combination of BMN-111 and LB-100 resulted in elongation during the culture period that was 16% greater than with BMN-111 alone.

We also measured the effect of LB-100 and BMN-111 on the increase of the total bone and cartilage area, defined as the area within the periphery of a photograph of the femur (Supplemental Figure 5). LB-100 and BMN-111 each individually increased this area, with a growth ratio of 1.40 for LB-100/control, and a growth ratio of 1.51 for BMN-111/control (Figure 3D). The combination of LB-100 and BMN-111 was even more effective, with a growth ratio of 1.93. Thus, the combination of BMN-111 and LB-100 enhanced the increase in bone and cartilage area by 27% compared with BMN-111 alone (Figure 3D).

### Combined treatment with LB-100 and BMN-111 improves growth plate cartilage homeostasis in Fgfr3^Y367C/+^ femurs.

**Figure 4:**
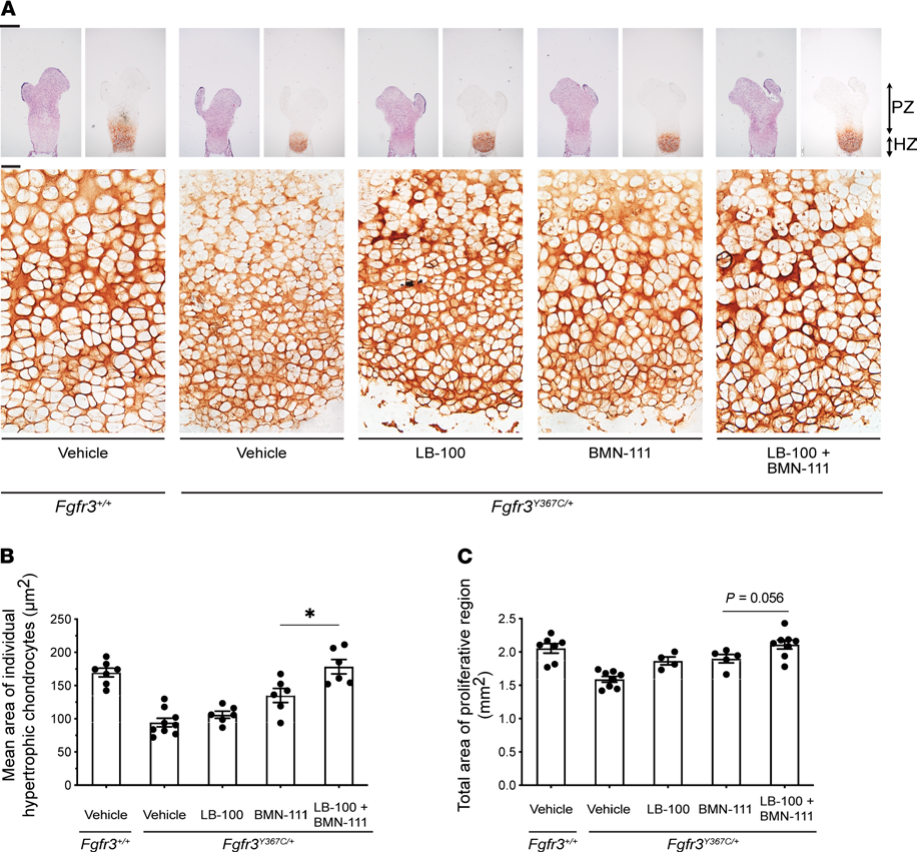
Dual action of LB-100 and BMN-111 improves chondrocyte differentiation and increases the proliferative growth plate area of ex vivo cultured *Fgfr3^Y367C/+^* femurs. (**A**) Representative images of HES-stained and COLX immunostained proximal growth plates of embryonic *Fgfr3^Y367C/+^* femurs incubated for 6 days with vehicle or LB-100 (10 μM) and/or BMN-111 (0.1 μM). Growth plates of *Fgfr3^+/+^* femurs cultured with vehicle are also shown. PZ, proliferative zone; HZ, hypertrophic zone. Scale bars: 400 μm (upper row), 20 μm (lower row). (**B**) Mean area of individual hypertrophic chondrocytes in proximal growth plates of femurs treated as described for **A** (*n =* 6–9 bones measured for each condition, with 54–185 cells measured for each bone). (**C**) Total proliferative area of the proximal plus distal growth plates of femurs treated as described for **A** (*n =* 4–8 bones measured for each condition). Symbols represent individual bones. Data are shown as mean ± SEM. Data were analyzed by 2-tailed unpaired *t* tests between the indicated groups. *P < 0.05.

Histological analyses of the epiphyseal growth plates of *Fgfr3^Y367C/+^* femurs showed that combining BMN-111 and LB-100 treatments improved cartilage growth homeostasis (Figure 4). Prehypertrophic and hypertrophic chondrocytes produce an extracellular matrix rich in Collagen type X (COLX); we used COLX immunostaining to label the hypertrophic region and to visualize and measure individual cells. This labeling revealed a highly beneficial effect of the combined treatment on the size of the cells in the hypertrophic area of *Fgfr3^Y367C/+^* femurs (Figure 4, A and B). The mean cross-sectional area of individual hypertrophic chondrocytes of *Fgfr3^Y367C/+^* mice was reduced by about half compared with that in the *Fgfr3^+/+^* growth plate (Figure 4, A and B; measured as described in Supplemental Figure 6). As previously reported (32), BMN-111 increased the size of the *Fgfr3^Y367C/+^* hypertrophic chondrocytes, but the cells remained smaller than for the WT (Figure 4, A and B). However, with the combined treatment of BMN-111 and LB-100, the mean area of the *Fgfr3^Y367C/+^* hypertrophic cells in the proximal growth plate was 32% greater than with BMN-111 alone and was similar to that of *Fgfr3^+/+^* hypertrophic cells, indicating that the final differentiation of the chondrocytes was restored by the treatment (Figure 4, A and B). Corresponding measurements for the distal growth plate showed a similar trend (Supplemental Figure 7).

We also observed a beneficial effect of the combined treatment on the proliferative region of the growth plate of *Fgfr3^Y367C/+^* mice. We measured the area of the proliferative region by subtracting the hypertrophic area, identified by COLX labeling, from the total growth plate area. Based on these measurements, the combined treatment increased the total proliferative growth plate area of the femur by an average of 33% over vehicle, compared with 20% for BMN-111 alone (Figure 4C). Thus, the combined treatment increased the proliferative area by 13% compared with BMN-111 alone (Figure 4C).

**Figure 5:**
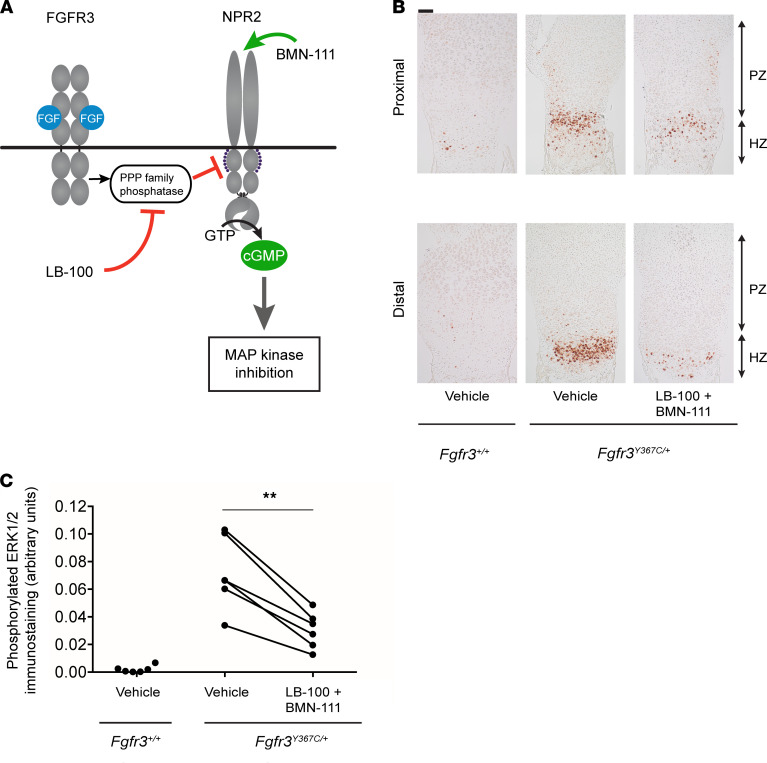
Dual action of LB-100 and BMN-111 decreases the activation of the MAP kinase pathway. (**A**) Diagram showing sites of action of LB-100 and BMN-111 on the MAP kinase pathway. (**B**) Representative images of phosphorylated ERK1/2 immunostaining of proximal and distal growth plates of embryonic *Fgfr3^Y367C/+^* femurs incubated for 6 days with vehicle or with LB-100 (10 μM) and BMN-111 (0.1 μM). Growth plates of WT (*Fgfr3^+/+^*) femurs cultured with vehicle are also shown. Scale bar: 100 μm. (**C**) Quantitation of phosphorylated ERK1/2 immunostaining in proximal plus distal growth plates of femurs from the same *Fgfr3^Y367C/+^* animal treated with either vehicle or a combination of LB-100 and BMN-111. Symbols represent individual femurs (*n =* 6 bones per condition). Data were analyzed by paired *t* test; **P < 0.01. Data from 6 vehicle-treated *Fgfr3*^+/+^ femurs are shown for comparison.

CNP signaling through NPR2 in the growth plate inhibits the MAP kinase pathway and its extracellular signal–regulated kinase 1 and 2 (ERK1/2) (refs. 31, 45, 46; Figure 5A). Therefore, we investigated the impact of treatment with LB-100 and BMN-111 on the phosphorylation of ERK1/2 in growth plates from *Fgfr3^Y367C/+^* embryos. As expected, immunolabeling showed a high level of phosphorylated ERK1/2 in the proximal and distal parts of the cartilage compared with WT controls (Figure 5, B and C). The combined LB-100 and BMN-111 treatment of *Fgfr3^Y367C/+^* femurs decreased the activity of the MAP kinase pathway, as demonstrated by the decreased phosphorylation of ERK1/2 in the proximal and distal growth plates of the femurs (Figure 5, B and C).

### The combination of LB-100 and BMN-111 enhances growth and improves chondrocyte differentiation in the ex vivo–cultured Fgfr3^Y367C/+^ skull base.

**Figure 6:**
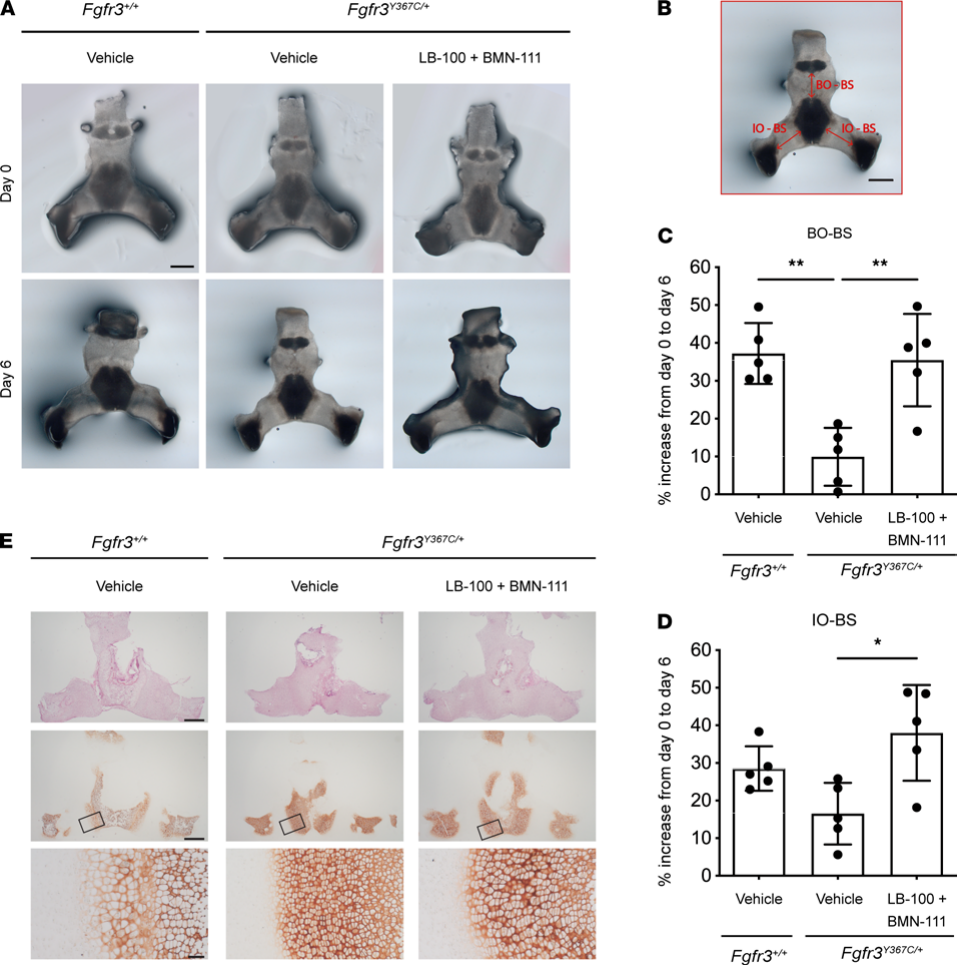
The combination of LB-100 and BMN-111 enhances growth and improves chondrocyte differentiation in the ex vivo cultured *Fgfr3^Y367C/+^* skull base. (**A**) Photographs of ex vivo cultures of skull bases from *Fgfr3*^+/+^ and *Fgfr3*^Y367C/+^ embryos before (day 0) and after a 6-day culture with the indicated treatments. The concentration of LB-100 was 10 μM, and the concentration of BMN-111 was 0.1 μM. (**B**) Diagram of the measurement positions for the graphs shown in **C** and **D**. BO, basioccipital; BS, basisphenoid; IO, interoccipital. Scale bars: 500 μm (**A** and **B**). (**C** and **D**) Percent increases in the indicated lengths between day 0 and day 6. Symbols represent individual skull base explants (*n =* 5). Data are shown as mean ± SEM. Data were analyzed by 1-way ANOVA followed by Tukey’s multiple comparison test. *P < 0.05, **P < 0.01. (**E**) HES-stained and COLX immunostained embryonic *Fgfr3^Y367C/+^* skull bases treated for 6 days with vehicle or LB-100 (10 μM) and BMN-111 (0.1 μM). Skull bases of *Fgfr3^+/+^* embryos cultured with vehicle are also shown. Representative images for each condition. Upper and middle rows, HES and COLX staining at low magnification; lower row, magnified view of COLX staining within the box marked in the middle row. Scale bars: 500 μm (upper and middle rows), 50 μm (lower row). Five samples for each condition.

Because compression of the spinal cord at the level of the foramen magnum (part of the skull base) is a critical clinical feature of ACH, contributing significantly to infant morbidity, we tested whether the combination of LB-100 and BMN-111 improves the defective growth of the skull base observed in *Fgfr3^Y367C/+^* mice. To investigate this question, we developed a model of ex vivo culture of the skull base isolated from mouse embryos (E16.5; Figure 6A). Over a 6-day culture period, we observed that the elongation of the cranial base was altered in explants from *Fgfr3^Y367C/+^* embryos compared with *Fgfr3^+/+^* embryos, because of a reduced size of the spheno-occipital and interoccipital synchondroses, localized respectively between the basioccipital bone (BO) and basisphenoid bone (BS), and between the interoccipital bone (IO) and BO (Figure 6, B–D).

The combination of LB-100 and BMN-111 increased the percent of growth of the 2 synchondroses in explants from *Fgfr3^Y367C/+^* embryos (Figure 6, C and D), leading to a rescue of the skull base anomalies, with similar bone elongation comparing *Fgfr3^+/+^* and treated *Fgfr3^Y367C/+^* explants (Figure 6, C and D). Histological analyses of the synchondrosis showed that combined BMN-111 and LB-100 treatments improved cartilage homeostasis in *Fgfr3^Y367C/+^* explants (Figure 6E). COLX immunolabeling revealed a highly beneficial effect of the combined treatment on the size of the cells in the hypertrophic area of *Fgfr3^Y367C/+^* cartilage.

## Discussion

Understanding of the mechanisms by which FGF/FGFR3 and CNP/NPR2 regulate longitudinal bone growth has allowed the development of an effective therapeutic strategy using a CNP analog (vosoritide; BMN-111) to treat ACH (33, 34). The findings described here identify the PPP-family phosphatase inhibitor LB-100 as a stimulator of bone growth when used in combination with this CNP analog to stimulate production of cGMP by NPR2. Firstly, using isolated WT bones incubated with FGF to mimic an ACH-like condition, we show that pretreatment with LB-100 counteracts the decrease in NPR2 guanylyl cyclase activity by FGFR3. Secondly, our results show that FGFR3 activation leads to NPR2 dephosphorylation in primary cultured WT chondrocytes and that LB-100 suppresses the dephosphorylation. Moreover, application of a combination of BMN-111 and LB-100 to long bones from the ACH mouse model *Fgfr3^Y367C/+^* results in growth that exceeds that stimulated by BMN-111 alone, and this combination also increases growth of the cranial base. This beneficial impact of the treatment on skull base elongation in *Fgfr3^Y367C/+^* mice and the correction of their defects are promising because the stenosis of the foramen magnum of ACH patients results from defective cranial base elongation. These results provide a proof of concept that BMN-111 and a PPP-family phosphatase inhibitor could potentially be used in combination for treatment of skeletal dysplasias such as ACH.

Our data also show the benefit of this treatment for growth plate cartilage during bone development in *Fgfr3^Y367C/+^* mice. During the process of endochondral ossification, chondrocytes actively proliferate in the resting and proliferating chondrocyte zone and then differentiate to hypertrophic chondrocytes, which lose the capacity to proliferate. The terminally differentiated hypertrophic cells are removed by cell death or transdifferentiate into osteoblasts. It is well known that FGFR3 signaling decreases bone growth by inhibiting both proliferation and differentiation of chondrocytes (47), and it has been proposed that FGFR3 acts by way of ERK1/2 to restrict hypertrophic differentiation (48). Here, we showed that treatment with BMN-111 and LB-100 reduced the levels of phosphorylated ERK1/2, thus modifying chondrocyte differentiation and allowing bone growth. In addition, we noted an impressive increase in the size of the hypertrophic cells. We concluded that the treatment restored cartilage homeostasis, and we hypothesize that the elevated cGMP resulting from this treatment could be a key regulator of transdifferentiation of hypertrophic cells into osteoblasts and could control the chondrogenic or osteogenic fate decision.

The increase in NPR2 phosphorylation by LB-100 is correlated with improved chondrocyte proliferation and differentiation in *Fgfr3^Y367C/+^* femurs, consistent with results with a mouse model (*Npr2^7E/7E^*) mimicking constitutive phosphorylation of NPR2 (27, 30). Because LB-100 inhibits multiple PPP-family phosphatases (35) (Table 1), and because its safety for long-term use in children is unknown, our results provide only a proof of principle for a possible combination treatment. Future studies to determine which phosphatases act to dephosphorylate NPR2 in chondrocytes are clearly warranted, and increased height in children with mutations in particular PPP2 regulatory subunit genes provides a clue (49). Identification of these phosphatases and development of more specific inhibitors targeting them could lead to future therapies.

Recent mouse studies indicate that, in addition to increasing prepubertal bone elongation, phosphorylation of NPR2 increases bone density, due to an increase in the number of active osteoblasts at the bone surface (50). Because low bone density is one of the key clinical features of ACH (51), the combination a CNP analog and a phosphatase inhibitor could also have a beneficial impact on bone density for patients with ACH and related conditions. In addition, such a treatment could have potential for treatment of osteoporosis and, because CNP/NPR2 also plays a key role in regulation of joint homeostasis, could be beneficial for preventing or minimizing cartilage loss and promoting repair of the damaged articular cartilage in skeletal disorders and osteoarthritis (52). More generally, the combination of natriuretic peptides and phosphatase inhibitors could have therapeutic potential for multiple disorders involving NPR2 and the related guanylyl cyclase NPR1 that also requires phosphorylation for activity (53).

In summary, the combined (LB-100 and BMN-111) treatment acts on both chondrocyte proliferation and differentiation, thus promoting better bone growth. In ACH, the homeostasis of the growth plate is disturbed, and proliferation and differentiation are affected by the overactivation of FGFR3. Currently, BMN-111 (vosoritide) is being studied in children with ACH and, as demonstrated in preclinical studies (32), mostly restores the defective differentiation in the growth plate. Recently reported phase 2 and 3 data demonstrate that BMN-111 results in a sustained increase in annualized growth velocity for up to 42 months in children 5–14 years of age with ACH (33, 34). The present study provides a proof of concept that a combination of BMN-111 and a phosphatase inhibitor has the potential to increase bone growth rate in ACH patients to a higher level than BMN-111 alone.

## Methods

### Mice.

Three mouse lines were used for this study: cGi500 (54), HA-Npr2 (43), and *Fgfr3^Y367C/+^* (44). The cGi500 mice were provided by Robert Feil (University of Tübingen, Tübingen, Germany). The strain for all mouse lines was CB7BL/6J; no sex selection was made. All experiments were performed using E16.5 embryos or 1- to 2-day-old newborns, as described for individual procedures.

### Reagents.

CNP and ANP were obtained from Phoenix Pharmaceutical (catalogs 012-03 and 005-24, respectively). BMN-111 was synthesized by New England Peptide as a custom order with the following sequence: (Cyc[23,39])H2N-PGQEHPNARKYKGANKKGLSKGCFGLKLDRIGSMSGLGC-OH, as previously described (32). The purity was > 95%. DEA/NO was from Cayman Chemical (catalog 82100). LB-100 (3-[4-methylpiperazine-1-carbonyl]-7-oxabicyclo[2.2.1]heptane-2-carboxylic acid) was from Selleck Chemicals (catalog S7537) or MedChem Express (catalog HY-18597). Cantharidin was from Tocris (catalog 1548). FGF18 was from PeproTech (catalog 100-28), and heparin was from Sigma-Aldrich (catalog H4784). DiFMUP (6,8-Difluoro-4-methyl-7-[phosphonooxy]-2H-1-benzopyran-2-one) was from Thermo Fisher Scientific (catalog D6567).

### Measurements of cGMP production in tibia growth plates using cGi500.

cGMP production in chondrocytes within intact growth plates was measured using tibias dissected from newborn mice (0- to 1-day-old mice) that globally expressed 1 or 2 copies of the cGi500 FRET sensor, as previously described (27). Tibias were dissected and cultured overnight on Millicell organotypic membranes (PICMORG50; MilliporeSigma) in BGJb medium (Thermo Fisher Scientific, catalog 12591-038) with 0.1% BSA (MP Biomedicals, catalog 103700), 100 units/mL of penicillin, and 100 μg/mL of streptomycin (Thermo Fisher Scientific, 15140-122). In preparation for imaging, each tibia was slit to remove the tissue overlying the growth plate. Where indicated, the tibia was incubated in LB-100, cantharidin, or control medium, followed by addition of FGF18 (0.5 μg/mL + 1 μg/mL heparin) or control medium containing heparin only. The tibia was then placed in a perfusion slide (ibidi USA, catalog 80186, special order with no adhesive), and the growth plate was imaged on the stage of a confocal microscope, as previously described (27).

### Determination of the effect of LB-100 on PPP1C phosphatase activity.

The coding sequence of human PPP1CA was expressed as a maltose binding protein fusion in a BL-21 strain of *E. coli* and purified as previously described (55). Phosphohistone phosphatase assays were performed as previously described (55, 56). Briefly, LB-100, at the indicated concentrations, or vehicle control (H_2_O) was added to enzyme/buffer aliquots about 10 minutes prior to starting assays by the addition of [^32^P]-phosphohistone substrate (to a final assay concentration of 300 nM incorporated phosphate). [^32^P]-phosphohistone was prepared by the phosphorylation of bovine brain histone (MilliporeSigma, type-2AS) with cAMP-dependent protein kinase (PKA) in the presence of cAMP and [^32^P]-ATP using established methods (56, 57). Phosphatase activity was measured by the quantitation of [^32^P]-labeled orthophosphate liberated from the substrate using established protocols (57). 6,8-Difluoro-4-methylumbelliferyl phosphate–based (DiFMUP-based) inhibition assays were conducted as described (56, 57), in a 96-well format using DiFMUP (Invitrogen) (100 μM final assay concentration). IC_50_ values were calculated from a 10-point concentration/dose response curve by a 4-parameter logistic fit of the data, using 3–8 replicates per concentration.

### Rib chondrocyte cultures.

Rib cages were dissected from newborn mice (0–2 days old) and trimmed to remove the skin, spinal cord, and soft tissue around the sternum and ribs. Nonchondrocyte tissue was digested away by incubating the rib cages in 2 mg/mL pronase (Roche, catalog 10165921001) in PBS for 1 hour in a shaking water bath at 37°C and was then incubated in 3 mg/mL collagenase D (Roche, catalog 11088866001) in medium for 1 hour. After washing, the rib cages were transferred to a dish with fresh collagenase D and incubated for 5–6 hours, with trituration at 2 hours, to release the chondrocytes. The isolated cells were passed through a 40 μm nylon cell strainer (Corning, catalog 431750), resuspended in DMEM/F12 medium (Thermo Fisher Scientific, catalog 11320-033) with 10% FBS (Thermo Fisher Scientific, catalog 10082-139), 100 units/mL of penicillin, and 100 μg/mL of streptomycin. The cells were plated in 35 mm tissue culture dishes, at a density corresponding to 1 newborn mouse per dish, and cultured for 3 days, at which point the cells were approximately 75%–90% confluent. They were then washed with PBS and incubated in serum-free medium for 18 hours. The cells were then incubated in LB-100 (10 μM), or control medium, followed by addition of FGF18 (0.5 μg/mL + 1 μg/mL heparin) or control medium containing heparin only.

At end of the incubation period, dishes were washed in PBS, and cells were lysed in 250 μL of 1% SDS containing 10 mM sodium fluoride, 1 μM microcystin-LR (Cayman Chemical, catalog 10007188), and protease inhibitor cocktail (Roche, catalog 04 693 159 001). Protein content was determined by a BCA assay (Pierce, catalog 23225). The protein yield per newborn mouse was approximately 200–300 μg.

### Phos-tag gel electrophoresis and Western blotting.

Proteins were separated in a Phos-tag–containing gel, as previously described (58), except that chondrocyte lysates (30 μg protein) were used without immunoprecipitation. Phos-tag and protein size markers were obtained from Fujifilm Wako Pure Chemical (catalogs AAL-107 and 230-02461, respectively). For these studies, we used mice with HA-tagged NPR2 (43), and blots were probed with an antibody against the HA tag (Cell Signaling Technology, catalog 2367, 1:1000 dilution). The specificity of this antibody is validated in Supplemental Figure 3. Note that molecular weight markers are only approximate for Phos-tag gels.

### Ex vivo culture of fetal femurs and skull base.

Femurs from E16.5 embryos were cultured ex vivo, as described previously (32, 47). The left femur was cultured in the presence of LB-100 (10 μM), BMN-111 (0.1 μM), or LB-100 (10 μM) + BMN-111 (0.1 μM) and was compared with the vehicle-treated right femur. The bone’s length was measured on day 0 (D0) and D6. Images were captured with an Olympus SZX12 stereo microscope and quantified using cellSens software (Olympus). The results were expressed as the increase in femur length or area (D6 – D0) in the presence or absence of LB-100, BMN-111, or LB-100 + BMN-111. Bone length and area were measured as shown in Supplemental Figure 5. To generate the graphs shown in Figure 3, the length or 2-dimensional area on D0 was subtracted from the length or area on D6 to calculate the amount of growth. These measurements of growth in drug-treated bones were divided by the mean values from corresponding measurements of control (vehicle-treated) bones; the graphs show the ratio of treated/control growth.

Embryonic skull base (E16.5) dissections were performed under an Olympus SZX12 stereo microscope and the skull bases (including the spheno-occipital and interoccipital synchondroses) were placed on top of 250 μL of Matrigel (BD Biosciences) in 24-well plates and cultured for 6 days in DMEM with antibiotics and 0.2% BSA (MilliporeSigma) supplemented with vehicle or LB-100 (10 μM) + BMN-111 (0.1 μM). The distances between the BS, BO, and IO bones were measured on D0 and D6 using cellSens software (Olympus). Percentage increases in BS-BO and BO-IO were calculated for each sample by comparing D0 and D6. The mean of the left and right BO-IO measurements were used to calculate the BO-IO increase. Five embryos were used for each group.

### Histology.

After a 6-day culture period, fetal femur (E16.5) explants were fixed in 4% paraformaldehyde, decalcified with EDTA (0.4M), and embedded in paraffin. Serial 5 μm sections were stained with hematoxylin‑eosin‑safran (HES) reagent, using standard protocols. For immunohistochemical assessment, sections were labeled with the following antibodies and a Dako Envision Kit: anti-COLX (BIOCYC, catalog N.2031501005; 1:50 dilution), and anti–phosphorylated ERK1‑2 (Thr180/Tyr182) (Cell Signaling Technology, catalog 4370; 1:100 dilution). Images were captured with an Olympus PD70-IX2-UCB microscope and quantified using cellSens software.

Mean areas of individual hypertrophic chondrocytes were measured from COLX-labeled sections, within a 166 μm wide × 76 μm high box positioned 50 μm from mineralization front (Supplemental Figure 6). The measurements were made manually using Fiji software and the freehand selection tool. For analysis of the effect of the drug treatments on the area occupied by proliferative chondrocytes, these cells were identified by their round or columnar shape, as seen with HES staining, and by the absence of COLX labeling. We measured the total area occupied by chondrocytes within the whole growth plate and the area occupied by COLX^+^ chondrocytes. The area for proliferating chondrocytes was calculated by subtracting the COLX^+^ area from the whole growth plate area.

### Statistics.

Data were analyzed using Prism 6 (GraphPad Software). To compare more than 2 groups, we used 1-way ANOVA followed by 2-tailed *t* tests with the Holm-Sidak correction for multiple comparisons, or 2-way ANOVA followed by Sidak’s multiple comparisons tests. Two groups were compared using either paired or unpaired 2-tailed *t* tests, as indicated in the figure legends.

### Study approval.

All experiments were conducted as approved by the animal care committees of the University of Connecticut Health Center and the Imagine Institute, Université de Paris.

## Author contributions

LCS, LAJ, and LLM designed the research and wrote the paper. NK performed the ex vivo bone growth experiments. MBD developed and analyzed the ex vivo skull base experiments. LCS and GV performed the cGMP imaging experiments. JRE, TFU, and TH performed the chondrocyte cell culture and Phos-tag analysis. LL and ED performed the immunolabeling quantitation. MRS and REH determined LB-100 selectivity for inhibition of PPP family phosphatases. LCS, NK, ED, MRS, MBD, and JRE prepared the figures.

## Supplementary Material

Supplemental data

## Figures and Tables

**Table 1:**
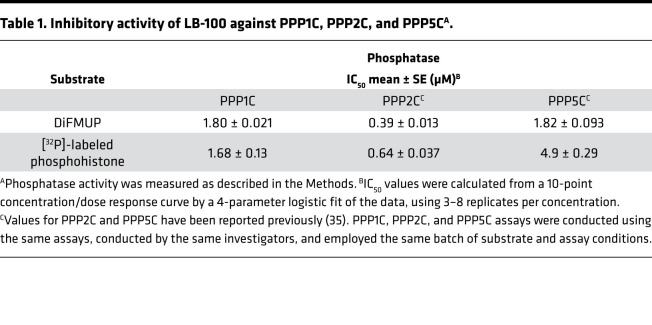
Inhibitory activity of LB-100 against PPP1C, PPP2C, and PPP5C^A^.

## Version 1 05/10/2021

Electronic publication
