# Supplementary material for: Phosphatase inhibition by LB-100 enhances BMN-111 stimulation of bone growth
Source: JCI Insight. 2021 May 10;6(9):e141426. doi: 10.1172/jci.insight.141426 (PMC8262325; doi:10.1172/jci.insight.141426)

SUPPLEMENTAL FIGURES

Phosphatase inhibition by LB-100 enhances BMN-111 stimulation of bone growth

Leia C. Shuhaibar[1], Nabil Kaci[2, 3], Jeremy R. Egbert[1], Thibault Horville[2], Léa Loisay[2], Giulia Vigone[1], Tracy F. Uliasz[1], Emilie Dambroise[2], Mark R. Swingle[4], Richard E. Honkanen[4], Martin Biosse Duplan[2, 5], Laurinda A. Jaffe[1*], Laurence Legeai-Mallet[2*]

Figure S1. Like CNP, BMN-111 stimulates cGMP production in growth plate chondrocytes. Mean ± SEM for 2 experiments with 0.1 μM BMN-111, and 2 experiments with 0.1 μM CNP, using tibias from littermates.

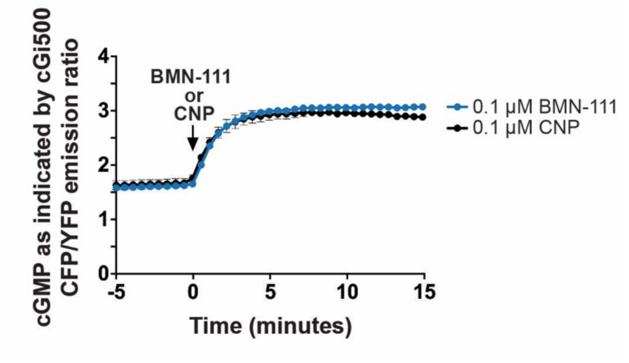

Figure S2. CNP increases cGMP production in growth plate chondrocytes, but ANP and DEA/NO do not. Graphs show the time courses of the CFP/YFP emission ratio from cGi500 after each agonist was perfused across the growth plate. (**A**) 0.1 μM CNP. Mean ± SEM for 27 similar experiments (trace from ***Figure 1B***). (**B**) 0.1 μM ANP. Mean ± SEM for 2 similar experiments. (**C**) 10 μM DEA/NO. Mean ± SEM for 3 similar experiments.

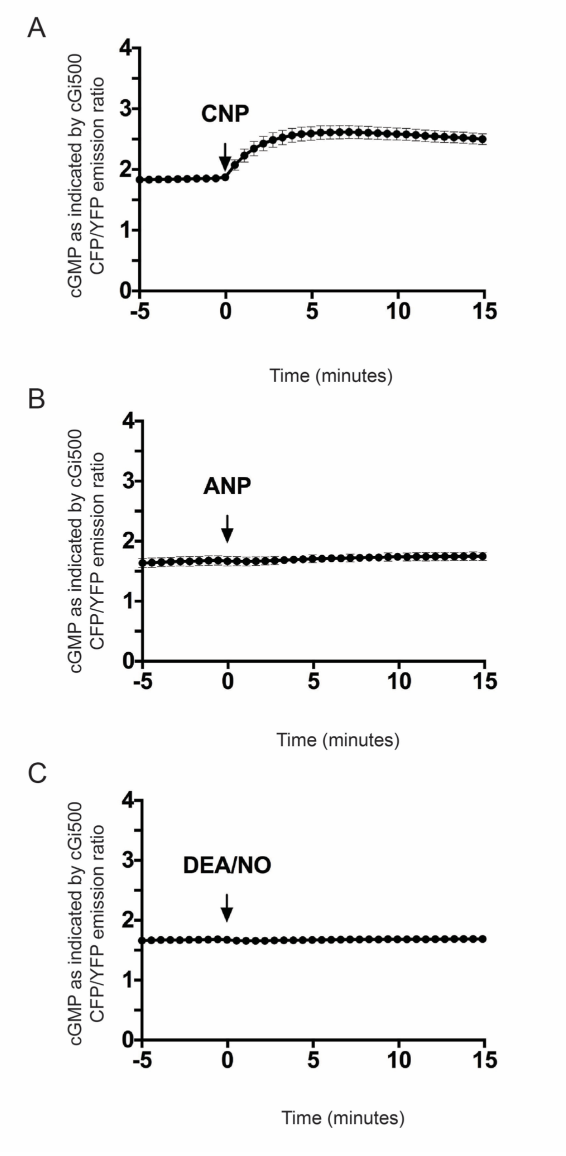

Figure S3. Specific detection of HA-NPR2 protein in chondrocytes from mice in which endogenous *Npr2* is tagged with an HA epitope. Western blot of a standard SDS-PAGE gel of 20 μg of protein from HA-*Npr2* and wildtype chondrocytes, probed with an antibody recognizing the HA epitope (Cell Signaling #2367). The two bands at ~120-130 kDa correspond to NPR2 with different amounts of glycosylation (Shuhaibar et al., 2016). No signal is detected in the wildtype control.

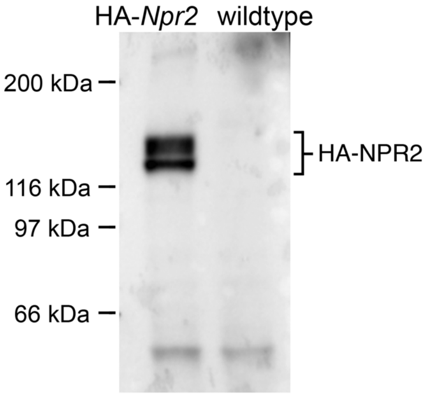

Shuhaibar LC, et al. Dephosphorylation of juxtamembrane serines and threonines of the NPR2 guanylyl cyclase is required for rapid resumption of oocyte meiosis in response to luteinizing hormone. *Dev Biol.* 2016;409(1):194-201.

Figure S4. Images of chondrocytes isolated from ribs of newborn mice. The chondrocytes were cultured for 4 days and then treated without (**A**) or with (**B**) LB-100 (10 μM) for 1 hour. Scale bars = 100 μm.

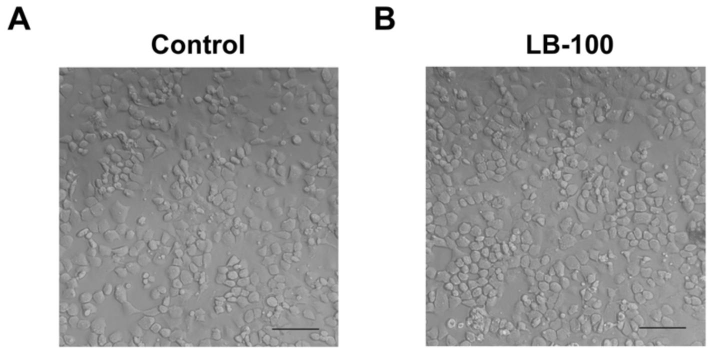

Figure S5. Regions of measurement for bone length and area. The photograph shows a fetal femur from a 16.5 day old *Fgfr3*$^{Y367C/+}$ mouse, after 6 days in culture. Bone length was defined as the sum of the lengths of the proximal epiphysis, the calcified ossification center (diaphysis), and the distal epiphysis (blue lines). Bone area was defined as the area within the red line. Scale bar = 500 μm.

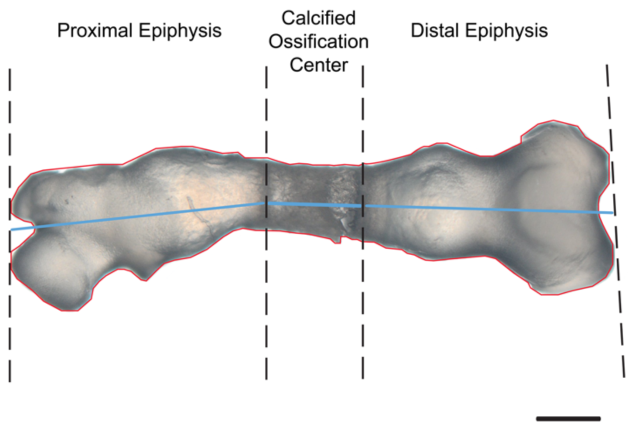

Figure S6. Region used to measure individual hypertrophic chondrocyte areas. A box 166 μm wide x 76 μm high was positioned at 50 μm above the mineralization front. Scale bars: 400 μm for upper image, 20 μm for lower image. Images are from ***Figure 4A***.

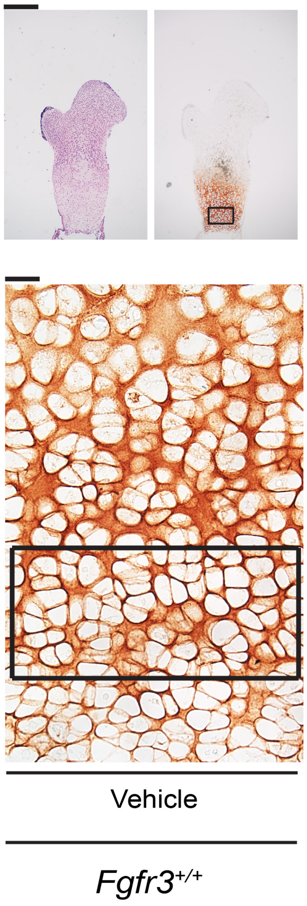

Figure S7. Mean area of individual hypertrophic chondrocytes in distal growth plates of femurs treated as described in ***Figure 4*** (n = 6-9 bones measured for each condition, with 40-149 cells measured for each bone). Symbols represent individual bones. Bars represent mean ± SEM. Data were analyzed by a two-tailed unpaired t-test between the indicated groups.

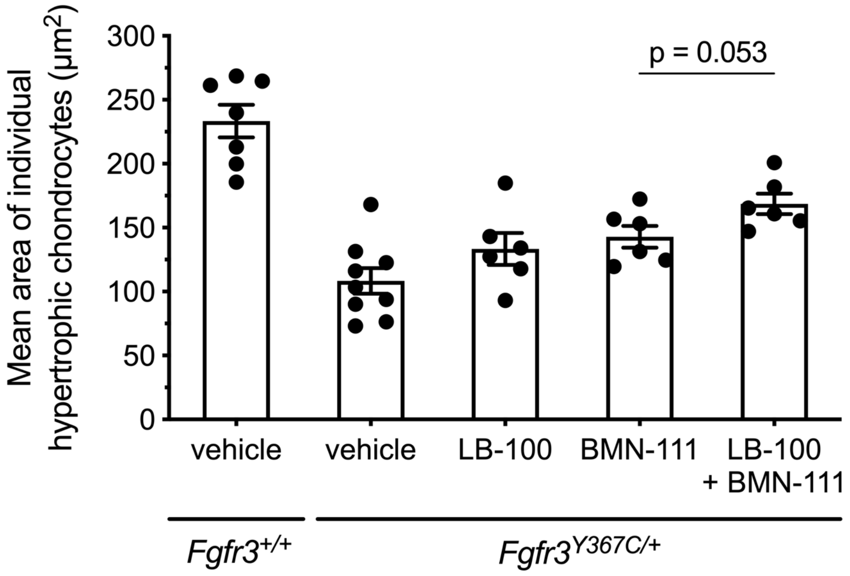

Supplement: Supplemental data [file jciinsight-6-141426-s095.pdf]
